# Visceral obesity is a preoperative risk factor for postoperative ileus after surgery for colorectal cancer: Single‐institution retrospective analysis

**DOI:** 10.1002/ags3.12291

**Published:** 2019-10-16

**Authors:** Yoshihiro Morimoto, Hidekazu Takahashi, Makoto Fujii, Norikatsu Miyoshi, Mamoru Uemura, Chu Matsuda, Hirofumi Yamamoto, Tsunekazu Mizushima, Masaki Mori, Yuichiro Doki

**Affiliations:** ^1^ Department of Gastroenterological Surgery Graduate School of Medicine Osaka University Osaka Japan; ^2^ Department of Mathematical Health Science Graduate School of Medicine Osaka University Osaka Japan; ^3^ Department of Therapeutics for Inflammatory Bowel Diseases Graduate School of Medicine Osaka University Osaka Japan; ^4^ Department of Surgery Graduate School of Medical Sciences Kyushu University Fukuoka City Japan

**Keywords:** colorectal cancer, ileus, obesity, postoperative complication, surgery

## Abstract

**Aim:**

Visceral obesity (VO) reportedly has a stronger association with complications after colorectal surgery than does body mass index. Here, we retrospectively assessed VO as a risk factor for postoperative ileus (POI) after colorectal resection in patients with colorectal cancer.

**Methods:**

This study included 417 consecutive patients with colorectal cancer who underwent elective surgery at our institute from January 2010 to December 2012. Visceral fat area (VFA) was calculated by image analysis software. VO was defined as VFA ≥100 cm^2^. We assessed 49 factors, including VO, comorbidities, surgical procedure, and postoperative complications. Data were analyzed using a propensity score‐matching strategy.

**Results:**

Postoperative ileus occurred in 18 patients (4.3%) from the entire cohort, and in 14 (5.5%) of the 256 matched patients. Multivariate analysis (n = 417 patients) showed that significant risk factors for POI included VO (OR 7.9, 95% confidence interval [CI] 1.9‐32.1, *P *=* *.004), open surgery (OR 6.4, 95% CI 1.6‐26.7, *P *=* *.010), and pelvic/intra‐abdominal abscess (OR 11.0, 95% CI 1.1‐110.2, *P *=* *.041). Propensity score matching showed two independent risk factors in the multivariate analysis: VO (OR 6.2, 95% CI 1.3‐30.4, *P *=* *.025) and open surgery (OR 9.1, 95% CI 2.0‐40.5, *P *=* *.004).

**Conclusion:**

Visceral obesity may be an independent risk factor for POI in patients with colorectal cancer.

## Abstract 2

Visceral obesity reportedly has a stronger association with complications after colorectal surgery than does body mass index. We retrospectively assessed visceral obesity as a risk factor for postoperative ileus (POI) after colorectal resection in patients with colorectal cancer. Visceral obesity may be an independent risk factor for POI in patients with colorectal cancer.

## Graphical abstract

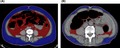

## 1 INTRODUCTION

Despite remarkable progress in the field of colorectal surgery, postoperative complications remain a major problem during the course of colorectal cancer treatment.^1^, ^2^, ^3^, ^4^ After colorectal surgery, postoperative ileus (POI) occurs at a frequency of 10%‐17%, resulting in longer hospital stays and higher costs.^3^, ^4^, ^5^, ^6^, ^7^ POI is characterized by lack of bowel sounds, delayed passage of flatus and stool, abdominal distension, nausea, vomiting, and pain.^5^, ^7^ Postoperative complications, including POI, after surgery for rectal cancer are reportedly associated with delays in adjuvant chemotherapy, and patients who receive delayed adjuvant chemotherapy have worse recurrence rates and worse overall survival than patients who receive chemotherapy within 8 weeks of surgery.^8^ Preoperative identification of risk factors could enable improved postoperative management for patients at higher risk of complications.

Prior studies have assessed the risk factors for POI, including male gender, peripheral vascular disease, respiratory comorbidity, preoperative albumin, stoma construction, operation lasting over 3 hours, conversion to open surgery, and intra‐abdominal surgical site infection.^3^, ^5^, ^9^ Compared to open surgery, laparoscopic colorectal surgery is associated with lower incidence of postoperative bowel obstruction.^10^ Some studies have reported that body mass index (BMI), which is widely used for the assessment of general obesity, is also a risk factor for POI.^6^, ^11^ However, recent findings suggest that, compared to BMI, visceral obesity (VO) is more strongly associated with complications after colorectal surgery.^12^, ^13^ In an analysis of 338 consecutive patients with colon cancer, Watanabe et al reported that VO was more strongly related to the incidence of anastomotic leakage and surgical site infection than high BMI.^13^ However, to the best of our knowledge, no study has shown that VO is a risk factor for POI after surgery for colorectal cancer.

In the present study, we assessed whether VO is a risk factor for POI among patients with primary colorectal cancer.

## 2 METHODS

### 2.1 Study population

**Figure 1:**
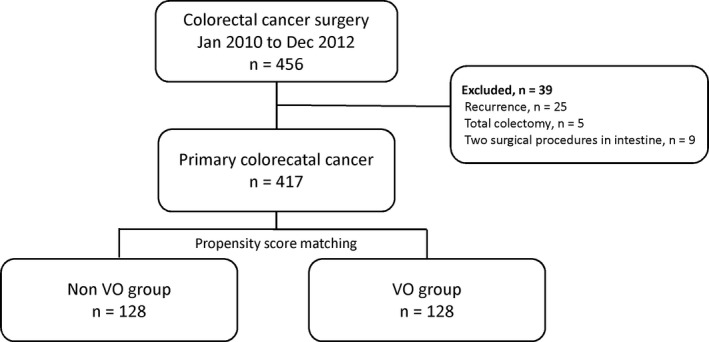
Flow diagram of patient inclusion in the present study. VO, visceral obesity

This study involved patients with primary colorectal cancer who underwent elective surgery at Osaka University Hospital between January 2010 and December 2012. We included patients who underwent one of 12 surgical procedures: ileocecal resection, right hemicolectomy, transverse colectomy, left hemicolectomy, sigmoidectomy, anterior resection, low anterior resection, super‐low anterior resection, abdominoperineal resection, intersphincteric resection, Hartmann's operation, or total pelvic exenteration. Patients who underwent subtotal colectomy, total colectomy, or two different procedures (eg, ileocecal resection and sigmoidectomy) were systematically excluded (Figure 1). All patients provided informed consent, and patient anonymity was preserved. This study was approved by the ethics committee at our institution.

### 2.2 Definitions

**Figure 2:**
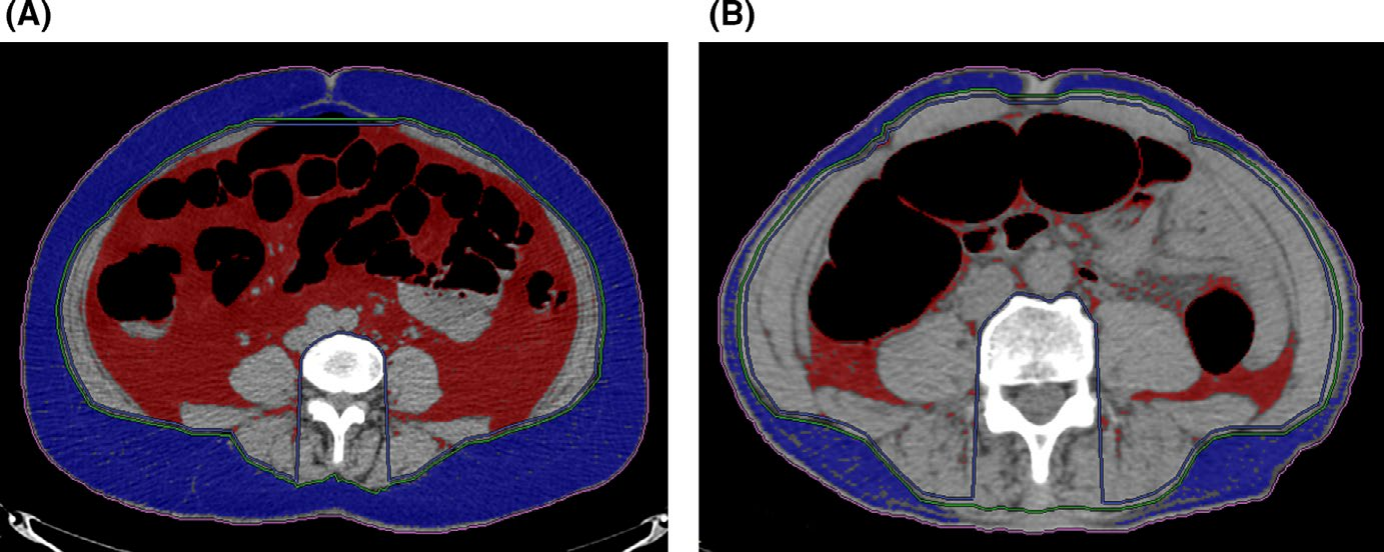
Images from the SYNAPSE VINCENT (Fuji Medical Systems, Tokyo, Japan) 3‐D image‐analysis system. Visceral fat is coloured in red, and its area was automatically calculated. A, Image from a patient with visceral obesity (visceral fat area = 231 cm^2^). B, Image from a patient without visceral obesity (visceral fat area = 23 cm^2^)

Postoperative ileus was defined as a Clavien‐Dindo grade II or higher ileus within 30 days after surgery. Ileus was diagnosed when patients complained of nausea, vomiting, or abdominal distension, and dilatation of the small bowel was radiologically confirmed without obvious small bowel obstruction.^14^ All patients underwent computed tomography (CT) prior to surgery, and visceral fat area (VFA) was calculated at the umbilicus level using the SYNAPSE VINCENT (Fuji Medical Systems, Tokyo, Japan) 3‐D image analysis system. Visceral fat, which has a Hounsfield unit threshold of −150 to −30, was coloured red in the images and its area calculated automatically (Figure 2). VFA ≥100 cm^2^ was considered to indicate VO.^12^, ^15^, ^16^

### 2.3 Data collection

We retrospectively collected demographic and clinicopathological data, including gender, age, BMI, VFA, comorbidities (cardiac comorbidity, neurological comorbidity, hypertension, pulmonary comorbidity, and diabetes mellitus), steroid use, American Society of Anesthesiologists (ASA) physical status classification, neoadjuvant chemotherapy, history of previous abdominal operation, preoperative serum albumin, tumor location, and pathological TNM stage. We also acquired retrospective data regarding the operation, including whether it was laparoscopic or open surgery, level of lymphadenectomy, type of surgical procedure (ie, ileocecal resection, right hemicolectomy, transverse colectomy, left hemicolectomy, sigmoidectomy, anterior resection, low anterior resection, super‐low anterior resection, abdominoperineal resection, intersphincteric resection, Hartmann's operation, or total pelvic exenteration), operative time, estimated blood loss, conversion to open surgery, resection of other organs, stoma construction, total intraoperative fluid, amount of intraoperative crystalloid, use of postoperative i.v. fentanyl, length of hospital stay, and postoperative complications (ie, ileus, intra‐abdominal haemorrhage, pelvic and intra‐abdominal abscess, anastomotic leakage, anastomotic hemorrhage, wound complications, urinary infection, and urinary retention).

### 2.4 Perioperative management

For mechanical bowel preparation the day before surgery, patients were given 2000 mL liquid containing polyethylene glycol in the evening, as well as two doses of 12 mg sennoside before bedtime. In the morning on the day of surgery, patients underwent an enema with 60 mL of 50% glycerine. Duration of preoperative fasting was 2 hours for liquids and 18‐24 hours for solids. Prophylaxis with i.v. antibiotics was given 5 minutes before the induction of anesthesia. During prolonged procedures, additional doses of antibiotics were given every 3 hours. To prevent deep vein thrombosis, all patients received mechanical thromboprophylaxis with well‐fitting compression stockings, and intermittent pneumatic compression was carried out during surgery and continued until the patient was fully ambulatory. The nasogastric tube was removed the day after surgery. Postoperative oral liquid intake was usually resumed the day after surgery, and a normal diet was resumed the third day after surgery. Patient mobilization was promoted the day after surgery.

### 2.5 Propensity score matching

We used a propensity score‐matching strategy to identify a population from which to estimate the number needed to harm for VO‐related bowel obstruction. To minimize confounding, we used patient demographics and VO risk to calculate propensity scores for VO as derived by a logistic regression model.

Propensity scores were derived using gender, age, comorbidities (ie, cardiac comorbidity, neurological comorbidity, hypertension, pulmonary comorbidity, and diabetes mellitus), steroid use, ASA score, neoadjuvant chemotherapy, history of previous abdominal operation, pathological TNM stage, laparoscopic or open surgery, and surgical procedure.

Propensity matching was carried out according to bowel obstruction risk using nearest‐neighbour matching without replacement, with each VO patient matched to one control patient. A caliper width of 0.2 standard deviation of the logit of the propensity score was used for the developed propensity score, and the same caliper width was used for the expected bowel obstruction and VO probability.

### 2.6 Statistical analysis

Demographic data were described across groups as the mean and standard deviation or median and range for continuous variables, and as the absolute count and proportion of patients for categorical variables. Student's *t*‐test was used for comparing quantitative variables, and Pearson χ^2^ test or Fisher's exact test was used to compare categorical data depending on sample size. Multivariate analysis for risk factors for POI was done using a logistic regression model including the variables with *P* values <.05 in the univariate analysis. Data were reported as the odds ratio (OR) and 95% confidence interval (CI). Significance was defined as a two‐sided *P* value <.05. All analyses were carried out using JMP Pro 13.1.0 (SAS Institute).

## 3 RESULTS

### 3.1 Clinicopathological characteristics of patients

A total of 417 patients were analyzed in this study. No patient died within 30 days after surgery. Table 1 summarizes the clinicopathological characteristics of all patients. Mean age was 64.9 years (SD 12.9 years), and 259 patients (62.1%) were male. Mean VFA was 88.7 cm^2^ (SD 48.0 cm^2^) and 156 patients (37.4%) met the criteria for VO. Cardiac comorbidities were seen in 64 patients (15.3%), neurological comorbidities in 18 (4.3%), hypertension in 114 (27.3%), pulmonary comorbidities in 19 (4.6%), and diabetes mellitus in 67 (16.1%).

**Table 1:** Clinicopathological characteristics of patients with colorectal cancer in the present study (n = 417)

Characteristic	
Gender	
Female	158 (37.9)
Male	259 (62.1)
Age, years	64.9 (SD 12.9)
Body mass index	22.5 (SD 3.4)
VFA, cm^2^	88.7 (SD 48.0)
Comorbidities	
Cardiac	64 (15.3)
Neurological	18 (4.3)
Hypertension	114 (27.3)
Pulmonary	19 (4.6)
Diabetes mellitus	67 (16.1)
Steroid use	9 (2.2)
ASA score	
1‐2	373 (89.4)
3‐4	44 (10.6)
Neoadjuvant chemotherapy	
Yes	19 (4.6)
No	398 (95.4)
Previous operation	
Yes	93 (22.3)
No	324 (77.7)
Preoperative serum albumin	3.8 (SD 0.5)
Tumor location	
Right‐sided colon	116 (27.8)
Left‐sided colon	129 (30.9)
Rectum	172 (41.2)
pT stage	
Tis‐T2	199 (47.7)
T3‐T4	218 (52.3)
pN stage	
N−	294 (70.5)
N+	123 (29.5)
Synchronous metastasis	
Yes	41 (9.8)
No	376 (90.2)

Note

Data are presented as n (%) or mean (standard deviation [SD]).

Abbreviations: ASA, American Society of Anesthesiologists; VFA, visceral fat area.

### 3.2 Surgical and postoperative outcomes

Table 2 presents the surgical and postoperative outcomes of all patients. POI occurred in 18 patients (4.3 per cent), and was treated with fasting and prokinetic agents in six patients, nasogastric tubes in eight patients, and long tube insertion in four patients. Two cases in which adhesiolysis and band dissection were carried out were excluded from the POI group. Median length of hospital stay was significantly longer for patients with POI (median 29 days, range 14‐157 days) than patients without POI (median 15 days, range 7‐223 days; *P *<* *.001).

**Table 2:** Surgical and postoperative outcomes of patients with colorectal cancer after colorectal resection (n = 417)

Laparoscopic surgery	
Yes	378 (90.6)
No	39 (9.4)
Lymphadenectomy	
D1	1 (0.2)
D2	113 (27.1)
D3	303 (72.7)
Surgical procedure	
Ileocecal resection	44 (10.6)
RHC	63 (15.1)
Transverse colectomy	9 (2.2)
LHC	31 (7.4)
Sigmoidectomy	98 (23.5)
AR	28 (6.7)
LAR	70 (16.8)
SLAR	36 (8.6)
APR	17 (4.1)
ISR	5 (1.2)
Hartmann's operation	6 (1.4)
Pelvic exenteration	10 (2.4)
Operative time, min	230 (71‐880)
Estimated blood loss, mL	50 (5‐11 150)
Conversion to open surgery	6 (1.4)
Resection of other organs	37 (8.9)
Stoma	52 (12.5)
Intraoperative crystalloid, mL	1730 (100‐6150)
Total intraoperative fluid, mL	2070 (300‐13 380)
Intravenous fentanyl	16 (3.8)
Complications	
POI	18 (4.3)
Intra‐abdominal haemorrhage	4 (1.0)
Pelvic/intra‐abdominal abscess	9 (2.2)
Anastomotic leakage	16 (3.8)
Anastomotic haemorrhage	12 (2.9)
Wound complication	20 (4.8)
Urinary infection	4 (1.0)
Urinary retention	4 (1.0)
Length of stay, days	15 (7‐223)

Note

Data are presented as n (%) or median (range).

Abbreviations: APR, abdominoperineal resection; AR, anterior resection; ISR, intersphincteric resection; LAR, low anterior resection; LHC, left hemicolectomy; POI, postoperative ileus; RHC, right hemicolectomy; SLAR, super‐low anterior resection.

### 3.3 Univariate and multivariate analyses of POI risk factors

Univariate analysis showed that patients with and without POI differed significantly in eight factors: VO, male gender, neoadjuvant chemotherapy, open surgery, sigmoidectomy, operative time >230 minutes, estimated blood loss >50 mL, and pelvic/intra‐abdominal abscess (Table 3). We carried out a multivariate analysis with seven of these factors, excluding sigmoidectomy due to the complete separation. Three factors remained significant independent risk factors for POI: VO (OR 7.9, 95% CI 1.9‐32.1, *P *=* *.004), open surgery, (OR 6.4, 95% CI 1.6‐26.7, *P *=* *.010) and pelvic/intra‐abdominal abscess (OR 11.0, 95% CI 1.1‐110.2, *P *=* *.041) (Table 3).

**Table 3:** Univariate and multivariate analyses for POI risk factors

	POI (n* *=* *18)	No POI (n* *=* *399)	Univariate analysis *P* value	Multivariate analysis	
				Odds ratio (95% CI)	*P* value
Male gender	16 (88.9)	243 (60.9)	**.01** **7**	4.1 (0.8‐21.9)	.091
Age, years	64.9 (SD 11.4)	65.9 (SD 11.9)	.737		
BMI ≥25	7 (38.9)	74 (18.6)	.060*		
VO	13 (72.2)	143 (35.8)	**.002**	**7.9 (1.9‐32.1)**	**.004**
Comorbidities					
Cardiac comorbidity	1 (5.6)	63 (15.8)	.331*		
Neurological comorbidity	0 (0)	18 (4.5)	1.000*		
Hypertension	6 (33.3)	108 (27.1)	.591*		
Pulmonary comorbidity	1 (5.6)	18 (4.5)	.576*		
Diabetes mellitus	4 (22.2)	63 (15.8)	.508*		
Steroid use	0 (0)	9 (2.3)	1.000*		
ASA score 3‐4	0 (0)	44 (11.0)	.238*		
Neoadjuvant chemotherapy	4 (22.2)	15 (3.8)	**.006** *	1.5 (0.2‐11.6)	.719
Previous operation	2 (11.1)	91 (22.8)	.385*		
Preoperative serum albumin	3.9 (SD 0.12)	3.8 (SD 0.03)	.424		
Tumor location					
Right‐sided colon	4 (22.2)	112 (28.1)	.588		
Rectum	11 (61.1)	161 (40.4)	.008		
pT3‐4 stage	10 (55.6)	208 (52.1)	.776		
pN+ stage	4 (22.2)	119 (29.8)	.604*		
Synchronous metastasis	4 (22.2)	37 (9.3)	.089*		
Open surgery	7 (38.9)	32 (8.0)	**<.001** *	**6.4 (1.6‐26.7)**	**.010**
D3 lymphadenectomy	14 (77.9)	289 (72.4)	.619		
Surgical procedure					
Ileocecal resection	1 (5.6)	43 (10.8)	.707*		
Right hemicolectomy	3 (16.7)	60 (15.0)	.743*		
Transverse colectomy	0 (0)	9 (2.3)	1.000*		
Left hemicolectomy	3 (16.7)	28 (7.0)	.142*		
Sigmoidectomy	0 (0)	98 (24.6)	**.010** *		
Anterior resection	3 (16.7)	25 (6.3)	.112*		
Low anterior resection	3 (16.7)	67 (16.8)	1.000*		
Super‐low anterior resection	2 (11.1)	34 (8.5)	.662*		
Abdominoperineal resection	0 (0)	17 (4.3)	1.000*		
Intersphincteric resection	0 (0)	3 (0.8)	1.000*		
Hartmann's operation	1 (5.6)	2 (0.5)	.124*		
Pelvic exenteration	2 (11.1)	8 (2.0)	.065*		
Operative time >230 min	14 (77.8)	194 (48.6)	**.016**	1.7 (0.4‐6.7)	.460
Estimated blood loss >50 mL	15 (83.3)	165 (41.4)	**<.001**	2.7 (0.6‐11.7)	.185
Conversion to open surgery	0 (0)	6 (1.5)	1.000*		
Resection of other organs	4 (22.2)	33 (8.3)	.065*		
Stoma	4 (22.2)	48 (12.0)	.261*		
Intraoperative crystalloid >1730 mL	12 (66.7)	195 (48.9)	.140		
Total intraoperative fluid >2070 mL	13 (72.2)	195 (48.9)	.053		
Intravenous fentanyl	2 (11.1)	14 (3.5)	.148*		
Complications					
Intra‐abdominal hemorrhage	1 (5.6)	3 (0.8)	.162*		
Pelvic/intra‐abdominal abscess	3 (16.7)	6 (1.5)	**.005** *	**11.0 (1.1‐110.2** **)**	**.041**
Anastomotic leakage	1 (5.6)	11 (2.8)	.415*		
Anastomotic hemorrhage	1 (5.9)	8 (2.0)	.424*		
Wound complication	0 (0)	20 (5.0)	1.000*		
Urinary infection	0 (0)	4 (1.0)	1.000*		
Urinary retention	1 (5.6)	3 (0.8)	.162*		

Note

Data are presented as n (%) or mean (standard deviation [SD]) unless otherwise noted.

Abbreviations: ASA, American Society of Anesthesiologists; BMI, body mass index; POI, postoperative ileus; VO, visceral obesity.

*Fisher's exact test. Bold text indicates a statistically significant difference with a *P*‐value less than 0.05.

### 3.4 Propensity score matching

To reduce the possibility of selection bias, we conducted propensity score matching. A total of 256 cases were matched (128 cases each). Table 4 presents their clinicopathological characteristics and preoperative features. Among all 417 patients, patients with and without VO had significant differences in gender, hypertension, diabetes mellitus, neoadjuvant chemotherapy, preoperative serum albumin, and synchronous metastasis. After matching, the two groups did not show any significant differences in clinicopathological characteristics and preoperative features (Table 4).

**Table 4:** Clinicopathological characteristics and preoperative features of patients

	All patients			Matched patients		
	VO (n* *=* *156)	no VO (n* *=* *261)	*P* value	VO (n* *=* *128)	no VO (n* *=* *128)	*P* value
Gender, male	119 (76.3)	140 (53.6)	**<.001**	92 (71.9)	93 (72.7)	.889
Age, years	65.4 (SD 1.0)	64.6 (SD 0.8)	.510	65.3 (SD 1.1)	65.5 (SD 1.1)	.881
Comorbidities						
Cardiac comorbidity	25 (16.0)	39 (14.9)	.767	18 (14.1)	21 (16.4)	.602
Neurological comorbidity	8 (5.1)	10 (3.8)	.528	7 (5.5)	7 (5.5)	1.000
Hypertension	57 (36.5)	57 (21.8)	**.001**	41 (32.0)	39 (30.5)	.787
Pulmonary comorbidity	6 (3.9)	13 (5.0)	.591	5 (3.9)	5 (3.9)	1.000
Diabetes mellitus	34 (21.8)	33 (12.6)	**.014**	25 (19.5)	22 (17.2)	.628
Steroid use	5 (3.2)	4 (1.5)	.304*	4 (3.1)	4 (3.1)	1.000*
ASA score (3‐4)	15 (9.6)	29 (11.1)	.631	11 (8.6)	10 (7.8)	.820
Neoadjuvant chemotherapy	3 (1.9)	16 (6.1)	**.046**	3 (2.3)	1 (0.8)	.622*
Previous operation	39 (25.0)	54 (20.7)	.306	30 (23.4)	25 (19.5)	.447
Preoperative serum albumin	3.9 (SD 0.04)	3.7 (SD 0.03)	**.013**	33.9 (SD 0.05)	3.8 (SD 0.05)	.165
Laparoscopic surgery	146 (93.6)	232 (88.9)	.111	117 (91.4)	118 (92.2)	.820
D3 lymphadenectomy	107 (68.6)	196 (75.1)	.149	87 (68.0)	85 (66.4)	.790
Surgical procedure						
Ileocecal resection	13 (8.3)	31 (11.9)	.254	11 (8.6)	13 (10.2)	.668
Right hemicolectomy	29 (18.6)	34 (13.0)	.125	25 (19.5)	21 (16.4)	.515
Transverse colectomy	5 (3.2)	4 (1.5)	.304*	3 (2.3)	2 (1.6)	1.000*
Left hemicolectomy	15 (9.6)	16 (6.1)	.189	11 (8.6)	13 (10.2)	.668
Sigmoidectomy	31 (19.9)	67 (25.7)	.177	28 (21.9)	29 (22.7)	.881
Anterior resection	10 (6.4)	18 (6.9)	.849	10 (7.8)	10 (7.8)	1.000
Low anterior resection	30 (19.2)	40 (15.3)	.302	23 (18.0)	22 (17.2)	.870
Super‐low anterior resection	15 (9.6)	21 (8.0)	.581	13 (10.2)	13 (10.2)	1.000
Abdominoperineal resection	4 (2.6)	13 (5.0)	.227	3 (2.3)	3 (2.3)	1.000*
Intersphincteric resection	0 (0)	3 (1.1)	.296*	0 (0)	0 (0)	
Hartmann's operation	0 (0)	3 (1.1)	.296*	0 (0)	0 (0)	
Pelvic exenteration	1 (0.6)	9 (3.4)	.098*	1 (0.8)	0 (0)	1.000*
pT (3‐4)	85 (54.5)	133 (51.0)	.485	65 (50.8)	66 (51.6)	.901
pN (+)	45 (28.8)	78 (29.9)	.822	37 (28.9)	37 (28.9)	1.000
Synchronous metastasis	22 (14.1)	19 (7.3)	**.024**	11 (8.6)	12 (9.4)	.827

Note

Data are presented as n (%) or mean (standard deviation [SD]).

Abbreviations: ASA, American Society of Anesthesiologists; VO, visceral obesity.

*Fisher's exact test. Bold text indicates a statistically significant difference with a *P*‐value less than 0.05.

### 3.5 Surgical and postoperative outcomes in matched patients

Table 5 shows the surgical and postoperative outcomes of the 256 matched patients. POI occurred in 14 (5.5 per cent) of the 256 matched patients and was treated with fasting and prokinetic agents in six patients, nasogastric tubes in seven patients, and long tube insertion in one patient. Median length of hospital stay was significantly longer among patients with POI (median 26 days, range 14‐50 days) than among patients without POI (median 15 days, range 8‐90 days; *P *=* *.012).

**Table 5:** Surgical and postoperative outcomes for matched patients (n = 256)

Laparoscopic surgery	
Yes	235 (91.8)
No	21 (8.2)
Lymphadenectomy	
D1	1 (0.4)
D2	83 (32.4)
D3	172 (67.2)
Operative time, min	228.5 (113‐729)
Estimated blood loss, mL	45 (5‐5660)
Conversion to open surgery	5 (2.0)
Resection of other organs	17 (6.6)
Stoma	24 (9.4)
Intraoperative crystalloid, mL	1750 (300‐6150)
Total intraoperative fluid, mL	2100 (300‐10 250)
Intravenous fentanyl	7 (2.7)
Complications	
POI	14 (5.5)
Intra‐abdominal hemorrhage	1 (0.4)
Pelvic/intra‐abdominal abscess	4 (1.6)
Anastomotic leakage	12 (4.7)
Anastomotic hemorrhage	8 (3.1)
Wound complication	13 (5.1)
Urinary infection	1 (0.4)
Urinary retention	2 (0.8)
Length of stay, days	15 (8‐90)

Note

Data are presented as median (range) or n (%).

Abbreviation: POI, postoperative ileus.

### 3.6 Univariate and multivariate analyses of POI risk factors in matched patients

Univariate analysis showed that patients with and without POI had significantly different rates of VO, open surgery, operation time >228.5 minutes, and estimated blood loss >50 mL (Table 6). Two of these factors, VO and open surgery, were also independent risk factors in the multivariate analysis: VO (OR 6.2, 95% CI 1.3‐30.4, *P *=* *.025), and open surgery (OR 9.1, 95% CI 2.0‐40.5, *P *=* *.004).

**Table 6:** Univariate and multivariate analyses of POI risk factors using matched patients

	Ileus (n* *=* *14)	No ileus (n* *=* *242)	Univariate analysis *P* value	Multivariate analysis	
				Odds ratio (95% CI)	*P* value
BMI ≥25	5 (35.7)	56 (23.1)	.332*		
Open surgery	5 (35.7)	16 (6.6)	**.003** *	**9.1 (2.0‐40.** **5)**	**.004**
D3 lymphadenectomy	10 (71.4)	162 (66.9)	1.000*		
Intra‐abdominal hemorrhage	0 (0)	1 (0.4)	1.000*		
Pelvic/intra‐abdominal abscess	1 (7.1)	3 (1.2)	.203*		
Anastomotic leakage	0 (0)	12 (5.0)	1.000*		
Anastomotic hemorrhage	0 (0)	8 (3.3)	1.000*		
Wound complication	0 (0)	13 (5.4)	1.000*		
Urinary infection	0 (0)	1 (0.4)	1.000*		
Urinary retention	0 (0)	2 (0.8)	1.000*		
Stoma	3 (21.4)	21 (8.7)	.132*		
Operation time >228.5 min	11 (78.6)	117 (48.3)	**.028**	3.5 (0.788‐16.0)	.099‐
Estimated blood loss >45 mL	12 (85.7)	116 (47.9)	**.006**	2.4 (0.460‐12.8)	.296
Conversion to open surgery	0 (0)	5 (2.1)	1.000*		
Resection of other organs	3 (21.4)	14 (5.8)	.056*		
Intraoperative crystalloid >1750 mL	9 (64.3)	114 (47.1)	.211		
Total intraoperative fluid >2100 mL	8 (57.1)	114 (47.1)	.465		
Intravenous fentanyl	1 (7.1)	6 (2.5)	.329*		
Visceral obesity	12 (85.7)	116 (47.9)	**.006**	**6.2 (1.3‐30.4** **)**	**.025**

Note

Data are presented as n (%) unless otherwise noted.

Abbreviations: BMI, body mass index; POI, postoperative ileus.

*Fisher's exact test. Bold text indicates a statistically significant difference with a p‐value less than 0.05.

## 4 DISCUSSION

Postoperative ileus is a frequent postoperative complication after colorectal surgery at our institution. POI is reported to result in more extended hospital stays and higher costs, as mentioned earlier. Therefore, we assessed the risk factors for POI in this setting. To effectively prevent POI, we must identify risk factors that can be assessed prior to surgery. In multivariate analysis including all 417 patients in our study cohort, we found that VO, open surgery, and pelvic/intra‐abdominal abscess are independent risk factors for POI. Of these, VO is a predictive factor that can be preoperatively assessed. Thus, we focused on analyzing VO as a risk factor for POI. As obesity is associated with both tumour initiation and progression in colorectal cancer,^17^, ^18^ it is commonly encountered among patients requiring colorectal surgery for colorectal cancer.

Previous reports have shown that, compared to BMI, VO is more strongly associated with complications after colorectal surgery.^12^, ^13^ Among 338 patients who underwent colorectal resection for colorectal cancer, and 75 patients who underwent total gastrectomy for gastric cancer, VO was more strongly related to incidences of anastomotic leakage and surgical site infection than high BMI.^13^, ^19^ However, to the best of our knowledge, no previous study has included multivariable analysis to assess VO as a risk factor for POI, and the present study is the first to show that VO may be a risk factor for POI after surgery for colorectal cancer.

Some studies have reported that BMI, which is widely used for the assessment of general obesity, is a risk factor for POI.^6^, ^11^ He et al. carried out a meta‐analysis, showing that BMI >30 is a risk factor for POI after laparoscopic colorectal surgery (OR 1.73, *P *=* *.02); however, they did not assess VO as a potential risk factor.^11^ In contrast, our present findings did not indicate that higher BMI is a risk factor for POI, although we used a cut‐off of 25 for higher BMI because only eight of our 417 patients had a BMI >30. This is compatible with other reports indicating that BMI is not a risk factor for POI after colorectal surgery.^3^, ^20^ In the present study, VO was an independent risk factor for POI, suggesting that VO is a more accurate predictor of POI after colorectal surgery than BMI. OR for VO and POI was 7.9 in the multivariate analysis, and 6.2 after propensity score matching, indicating that VO may have an impact on postoperative outcome as well as on POI.

Preoperative assessment of VO as a predictor of POI may enable action to be taken before surgery to prevent or reduce POI. Numerous strategies for reducing POI have been explored. In a systematic review, Chapman et al showed that minimally invasive surgery, protocol‐driven recovery (eg, early feeding and opioid‐avoidance strategies), and measures to avoid major inflammatory events (eg, anastomotic leakage) offer the best chances of reducing POI.^21^

Most reports on VO as a risk factor for postoperative complications do not discuss the mechanisms by which adipose tissue causes inflammation. Stoffels et al showed that interleukin‐1 (IL‐1) signaling by IL‐1 receptor 1 and myeloid differentiation primary response 88 (MyD88) is required for POI development after intestinal manipulation in mice.^22^ Studies of adipose tissue have shown that human visceral adipose tissue, which is considered a major contributor to increased levels of circulating inflammatory cytokines, contains higher levels of IL‐1α, IL‐1β, and IL‐1R antagonist compared to subcutaneous adipose tissue.^23^, ^24^, ^25^ Reasonably, IL‐1α and IL‐1β, which are both major components of the IL‐1 family, may be released from adipose tissue as a result of intraoperative manipulation of the intestines and mesentery, leading to the development of POI.

Median hospital stay in the present study was 14 days, even among patients without POI, although the length of hospital stay after surgery for elective colorectal cancer has been reported to be 4‐12 days.^21^, ^26^ Reason for the longer stay at our institution compared to current standards in most Western countries may be that we decide the date of discharge in part on the bed occupancy rate, as it is important for hospital management.^27^

Use of i.v. fentanyl after surgery was not a risk factor for POI in the present study, possibly because few patients were given i.v. fentanyl. We do not use epidural anesthesia or i.v. fentanyl after laparoscopic surgery for colorectal cancer unless we cannot control postoperative pain without opioids. Most of the patients (90.6%) underwent laparoscopic surgery, and i.v. fentanyl was given only to 16 (3.8%) of the 417 patients.

The present study has several limitations. First, the retrospective design of this study has several intrinsic limitations. To reduce the risk of selection bias, we carried out propensity score matching. Second, by the definition used for POI, there was a possibility of including patients with small bowel obstruction into the POI group although we excluded two cases with blatant small bowel obstruction. However, some perioperative interventions for patients with VO will be effective prevention for both POI and small bowel obstruction. Third, this study was carried out at a single institution with a moderate number of patients. Further studies should be conducted in larger populations from multiple institutions. Fourth, we did not assess postoperative levels of IL‐1 family members in the patients, but VO can be used as a surrogate marker of these levels. Fifth, the enhanced recovery after surgery (ERAS) protocol was not introduced at our institution. POI prevention is one of the aims of the ERAS protocol,^28^, ^29^ and Ni et al reported that ERAS resulted in shorter average length of postoperative hospital stay, time to first flatus, and time to first defecation than traditional perioperative care after colorectal surgery in 1298 patients in a meta‐analysis of randomized controlled trials.^29^ They also reported that the overall complication rates were significantly lower with ERAS than with traditional perioperative care. Although, currently, the ERAS protocol is not common in Japan, it may be effective, especially for patients with VO. Despite these limitations, we think that the present study is of great importance because it is the first report of VO as a risk factor for POI after surgery among patients with colorectal cancer.

In conclusion, the present study showed that VO is an independent risk factor for POI after colorectal resection among an entire cohort of 417 patients and among 256 matched patients. This finding enables the assessment of POI risk before surgery by checking VFA, as VFA >100 cm^2^ may indicate a need for perioperative interventions, such as opioid‐avoidance strategies and giving prokinetic agents before and after surgery. Furthermore, selectively introducing the ERAS protocol in Japan for patients with VO may be acceptable and effective. The present findings should be verified in a prospective multicenter study with a greater number of patients.

## DISCLOSURE

Conflicts of Interest: Authors declare no conflicts of interest for this article.

Author Contribution: All authors are in agreement with the content of the manuscript.
